# Moringa isothiocyanate-1 regulates Nrf2 and NF-κB pathway in response to LPS-driven sepsis and inflammation

**DOI:** 10.1371/journal.pone.0248691

**Published:** 2021-04-01

**Authors:** Badi Sri Sailaja, Rohit Aita, Shane Maledatu, David Ribnicky, Michael P. Verzi, Ilya Raskin

**Affiliations:** 1 Department of Plant Biology, School of Environmental and Biological Sciences, Rutgers, The State University of New Jersey, New Brunswick, New Jersey, United States of America; 2 Department of Genetics and the Human Genetics Institute of New Jersey, Rutgers, The State University of New Jersey, New Brunswick, New Jersey, United States of America; Augusta University, UNITED STATES

## Abstract

This study aims to document the dual mode of pharmacological action of moringa isothiocyanate-1 (MIC-1) derived from seeds of *Moringa oleifera* Lam. Oral administration of chemically stable MIC-1 (80 mg/kg) significantly reduced the expression of inflammatory markers (Tnf-α, Ifn-α, IL-1β, IL-6) in the liver, kidney, spleen, and colon and decreased spleen weight in the lipopolysaccharide (LPS)-induced sepsis / acute inflammation model in mice. Transcriptomic analysis of the effect of MIC-1 on the liver and in the LPS-induced RAW264.7 murine macrophage showed that MIC-1 decreases inflammation via inflammation, immunity, and oxidative stress pathways. These results are supported by the immunocytochemical observations that MIC-1 increased the nuclear accumulation of nuclear factor (erythroid-derived 2)-like 2 (Nrf2) transcription factor and decreased the nuclear accumulation of nuclear factor kappa-light-chain-enhancer of activated B cells (NF-κB) in the LPS-induced macrophages. Transcriptional activation of antioxidant genes by MIC-1 translated into a reduction of reactive oxygen species (ROS) in the cytoplasm, decrease of mitochondrial superoxide content, and restoration of the mitochondrial membrane potential in the LPS-induced macrophages. Our data indicate that MIC-1 affects inflammation and oxidative stress, two key processes involved in the etiology of many chronic diseases. These effects involve upstream regulation of two key transcriptional factors regulating responses to these processes at a gene expression level.

## Introduction

A state of inflammation is central to the pathophysiology of multiple chronic and communicable diseases and acute disorders, such as arthritis, inflammatory bowel disease, diabetes, cancer, injury, infections, and sepsis [1]. For thousands of years, traditional medicine has used plants to treat these conditions. In the last century, several natural products with strong anti-inflammatory properties, such as isothiocyanates (ITCs) present in several plant families, most notably Brassicaceae (e.g., cabbage, broccoli, mustards) and Moringaceae [2], have been isolated from plants [3]. Moringa (*Moringa oleifera* Lam.), a member of the Moringaceae family, is an edible and medicinal plant cultivated throughout the tropics [4]. Seeds and, to a lesser extent, leaves of moringa are rich in stable moringa isothiocyanates (MICs) [5, 6]. In contrast to most Brassicaceae ITCs, MICs are stable solids because of ring glycosylation [3, 6, 7].

Moringa isothiocyanate—1 (MIC-1) is the predominant isothiocyanate from moringa seeds. Through a process of seed grinding, water soaking, and ethanol extraction, a moringa seed extract (MSE) containing 35–45% of MIC-1 (w/w) can be produced, from which MIC-1 can be purified [6, 7]. MSE, as well as MICs-enriched moringa leaf extract, can mitigate obesity-related metabolic dysfunction in mouse intervention studies [7–10]. MSE also attenuate ulcerative colitis symptoms in mice [7], while showing relatively low toxicity to mice [11]. ITCs from Brassicaceae, such as sulforaphane, the main ITC from broccoli, have a number of health benefits that confer anti-inflammatory, chemoprotective, and anti-oxidant effects [12, 13]. The benefits of sulforaphane are linked to the activation of the nuclear factor (erythroid-derived 2)-like 2 (Nrf2) transcription factor, which translocates into the nucleus and promotes the transcription of genes required for protection from oxidative stress, detoxification of xenobiotics, maintenance of redox potential, and anti-inflammatory responses [14, 15]. Nrf2 affects transcription by binding to the antioxidant response element (ARE) in the promoter regions of its target genes [15, 16]. We previously demonstrated that at least some of the Nrf2-activated genes are also activated by MICs [7, 8, 17]. Sulforaphane may also inhibit nuclear factor kappa-light-chain-enhancer of activated B cells (NF-κB) [18], which may partially explain the anti-inflammatory properties of many ITCs. MICs may inhibit NF-κB activity in human carcinoma cells more effectively than sulforaphane [19].

MSE and its active component, MIC-1, strongly decreased inflammatory and oxidative stress markers in lipopolysaccharide (LPS)-induced mouse macrophages [7]. LPS, derived from Gram-negative bacteria, is a powerful inducer of inflammation, endotoxemia, and sepsis in cells and animals [20]. These pathological processes are closely associated with the production of reactive oxygen species (ROS) in the cytoplasm and organelles, primarily mitochondria [21]. Overproduction of mitochondrial superoxide leads to mitochondria damage and dysfunction [22], which is often observed during sepsis, which is a state of acute inflammation and runaway immune response [23, 24]. Despite the previous research into inflammatory and oxidative stress pathways, the molecular mechanisms by which MIC-1 and Brassicaceae ITCs acts to decrease inflammation and oxidative stress are still poorly understood.

Since MICs could mitigate many inflammatory and oxidative processes closely associated with sepsis and acute inflammation, we evaluated the effects of MIC-1 on the LPS-induced mouse sepsis model and investigated molecular mechanisms underlying its wide-ranging and often overlapping anti-inflammatory and anti-oxidative stress activities. The data confirmed our hypothesis that MIC-1 affects nuclear translocation of two key transcription factors involved in anti-oxidative stress and anti-inflammatory cellular defenses—Nrf2 and NF-κB.

## Materials and methods

### Chemicals and reagents

Lipopolysaccharide (LPS, *Escherichia coli* 0111: B4, Sigma). Dulbecco’s modified Eagle’s medium (DMEM), penicillin G, streptomycin, and fetal bovine serum (FBS) from Gibco Inc. (Grand Island, NY).

### Isolation and purification of MIC-1 from MSE

MIC-1 (purity >99%) was prepared as previously reported [6, 7]. Ground moringa seeds were incubated in water at a 1:3 ratio for 2 h at 37°C. Subsequently, ethanol was added at four times the volume of water and then filtered and dried through a rotary evaporator and freeze-dryer. To purify MIC-1, freeze-dried MSE was resuspended in ethanol (200 mg/mL) and sonicated for 30 min. The resuspended extract was then filtered using a 0.2 μm filter before injection in the HPLC. Using a semi-preparative reversed-phase high-performance liquid chromatography system equipped with an ultraviolet detector (HPLC-UV; Waters) monitored at 222 nm, MIC-1 prepared from the filtered extract was eluted using a gradient with initial conditions of 70% solvent A and 30% solvent B for 5 min. Solvent B was increased to 100% over 25 min and maintained for 5 min, returning to initial conditions over 2 min with an 8 min equilibration between injections. MIC-1 was collected, dried by rotary evaporation and subsequent lyophilization. The freeze-dried material was stored at -20°C. MIC-1 was resuspended in ethanol and filtered through a 0.2 μm syringe filter before injection (1 μL) into the LC-MS. Confirmation of MIC-1 identity based on the comparison of retention time, UV spectrum, and MS data of previously prepared standard materials [6].

### Cell culture

RAW264.7 cells (murine macrophage cell line, ATCC, Manassas, VA) were cultured in endotoxin-free DMEM supplemented with 10% FBS (fetal bovine serum), 100 U/mL penicillin, and 100 μg/mL streptomycin. THP-1 (Human monocytes) cell line were cultured in ATCC-formulated RPMI-1640 Medium, Catalog No. 30–2001. To make the complete growth medium, 0.05 mM 2-mercaptoethanol, 10% FBS was added. Cells were incubated at 37°C in a humidified atmosphere containing 5% CO_2_.

### Animals and treatment

C57BL/6 male mice were purchased from Charles River Laboratories (Malvern, PA) and acclimated for one week at 22 ± 2°C in a light/dark cycle of 12 h. Mice were housed two per cage and allowed access to food and water *ad libitum*. Experiments were performed using the approved protocol by the Rutgers University Institutional Animal Care and Use Committee. C57BL/6 male mice were randomly divided into three groups of six animals as follows: Group a: vehicle control group injected with saline; Group b: LPS (10 mg/kg); Group c: MIC-1 (80 mg/kg) gavaged group injected with LPS. Mice were pretreated for 3 d with MIC-1 in 10% DMSO (Sigma-Aldrich, Darmstadt, Germany) via oral gavage before an LPS intraperitoneal injection and then sacrificed at 16 h after treatment for tissue collection.

### Tissue isolation, blood collection, and preparation

At the time of sacrifice, the spleen, liver, kidney, and colon were dissected from each mouse. Individual spleen tissues were weighed (mg). The colons were thoroughly flushed with cold PBS (pH 7.4; supplemented with 1% antibiotics) to remove feces and blood. After pushing out the remaining colon contents with blunt forceps, the colon was opened by longitudinal incision and washed three times in cold PBS (pH 7.4; supplemented with 1% antibiotics) for complete removal of fecal matter, which was used for quantification of gene expression. Blood was collected from each mouse after cardiac puncturing for ELISA.

### Immunofluorescence

For immunofluorescence, RAW264.7 macrophage cells were grown on coverslips, fixed in 4% PFA (15 min, at room temperature, RT), washed (three times) in PBS (5 min, RT), permeabilized (0.5% Triton X-100, 5 min, RT), and incubated for 1 h at RT or 4°C overnight with the primary rabbit polyclonal antibodies Nrf2 (ab 62352) and NF-κB (ab 16502). After incubation, the cells were washed three times in PBS for 5 min at RT, incubated for 1 h at RT with secondary antibodies, washed again, and stained with phalloidin actin (Invitrogen, Carlsbad CA,) and DAPI for 5 min at RT. Imaging was done with the Zeiss LSM 710 Confocal Microscope.

### ELISA

Immunoreactive mouse Tnf-α and mouse IL-6 were quantified using commercially available ELISA kits (R&DSystems, Minneapolis, MN) according to the manufacturer’s instructions.

### RNA sequencing and analysis

RNA was extracted according to the manufacturer’s protocol with the RNeasy Mini Kit. (Qiagen) according to the manufacturer’s protocol and raw sequencing reads (fastq) files were obtained from BGI sequencing. Quantification for the transcript abundances of the RNA-seq samples was performed using a RefSeq transcriptome index for mm9 and through pseudoalignment was done using Kallisto v0.45.0 [25, 26]. The tximport v 1.8.0 [27] package was run in R v3.5.2 to create gene-level count matrices for use with DESeq2 v1.20 [28] by importing quantification data obtained from Kallisto, followed by the use of DESeq2 to generate FPKM values and to call genes as differentially expressed. Genes with FPKM>1, a commonly used minimal expression threshold, were used for further analysis. The Morpheus heat mapping software from the Broad Institute (https://software.broadinstitute.org/morpheus). was then used to display the relative transcription levels of the genes of interest by using normalized FPKM values. Gene Ontology analysis was performed to functionally annotate biological processes using DAVID v6.8 [29]. All RNA-seq data of this study have been deposited in GEO (GSE155738).

### RNA extraction and quantitative real-time PCR

Quantitative real-time PCR was performed using a Bio-Rad sequence detection system with a 15 μL reaction mixture containing 0.5 μM forward and reverse primers, Power SYBR Green PCR Master Mix (Applied Biosystems), and template cDNA. GAPDH was used as an internal control amplified in the same PCR assay. The quantitative PCR primers used in this study are listed in S1 Table.

### Chromatin immunoprecipitation

ChIP assays were performed primarily as previously reported [30, 31]. The chromatin solution was precleared with salmon sperm DNA/protein A-agarose 50% gel slurry (Millipore, Burlington MA) for 45 min at 4°C. The solution was then incubated with the 10 μg NF-κB antibody (ab16502) overnight at 4°C. As a control, samples were immunoprecipitated with nonimmune rabbit IgG (Millipore). After immunoprecipitation, the DNA–protein complex was collected with 60 μL protein A–agarose beads for 1 h. The beads were sequentially washed once with low salt, high salt, and LiCl, then washed twice with 10 mM Tris (pH 8)/1 mM EDTA buffers. The DNA–protein complex was eluted from the beads with a 250 μL elution buffer (1% SDS and 0.1 M NaHCO3). DNA and protein complex were reverse cross-linked at 65°C for 4 hours in high-salt conditions. Proteins were digested using proteinase K treatment for 1 h at 45°C. The DNA was extracted with phenol/chloroform/isoamyl alcohol, precipitated with 70% ethanol, and finally resuspended in 80 μL PCR-grade water. The ChIP quantitative PCR primers used in this study are listed in S1 Table.

### Determination of intracellular reactive oxygen species

The intracellular ROS was determined by the fluorescence of 2’, 7’-dichlorofluorescein (DCFH2-DA) [32]. Macrophage cells were seeded into 24-well cell culture plates at a concentration of 5 ×10^4^ cells/well and cultured for 24 h. Thereafter, cells were treated with MIC-1 (10 μM) along with and without lipopolysaccharide (LPS) (1 μg/mL) for 24 hours. Subsequently, cells were washed with PBS, incubated with 10 μM DCFH-DA at 37°C for 30 min, washed with PBS again, and imaged with a fluorescence microscope (FSX100, Olympus). ImageJ software was used for quantification for mean fluorescence intensity.

### Determination of mitochondrial superoxide

The mitochondrial superoxide level was determined by the fluorescence of the MitoSOX Red fluorescence stain [33]. Macrophage cells were seeded into 24-well cell culture plates at a concentration of 5 × 10^4^ cells/well and cultured for 24 hours. After that, cells were treated with MIC-1 (5 μM & 10 μM)) or MIC-1 and LPS (1 μg/mL) for 24 hours, washed with PBS, incubated with 5 μM MitoSOX Red for 30 min, and then again washed twice with PBS. After washing with PBS, the fluorescence intensity of the cells was visualized with fluorescence microscopy. ImageJ software was used to quantitate mean fluorescence intensity.

### Detection of mitochondrial membrane potential

Mitochondrial membrane potential (MMP) was determined with the fluorescent dye Rh-123 staining method, as reported earlier [34]. Macrophage cells were seeded into 24-well cell culture plates at a concentration of 5 × 10^4^ cells/well and cultured for 24 h. After that, cells were treated with MIC-1 (10 μM) with or without LPS (1 μg/mL) for 24 h. Cells were washed with PBS and stained with 10 μM of Rh-123 at 37°C for 30 min in the dark. After washing with PBS, the fluorescence intensity of the cells was visualized with fluorescence microscopy. The ImageJ software was used to quantitate mean fluorescence intensity.

### Statistical analysis

The results are expressed as mean standard deviation (SD) of three experiments. Significant differences in the mean values were evaluated by one-way analysis of variance or Student’s t-test using GraphPad (GraphPad, La Jolla, CA). Differences were considered statistically significant at p < 0.05.

## Results

### Transcriptome analysis of MIC-1 effects in macrophages

**Fig 1:**
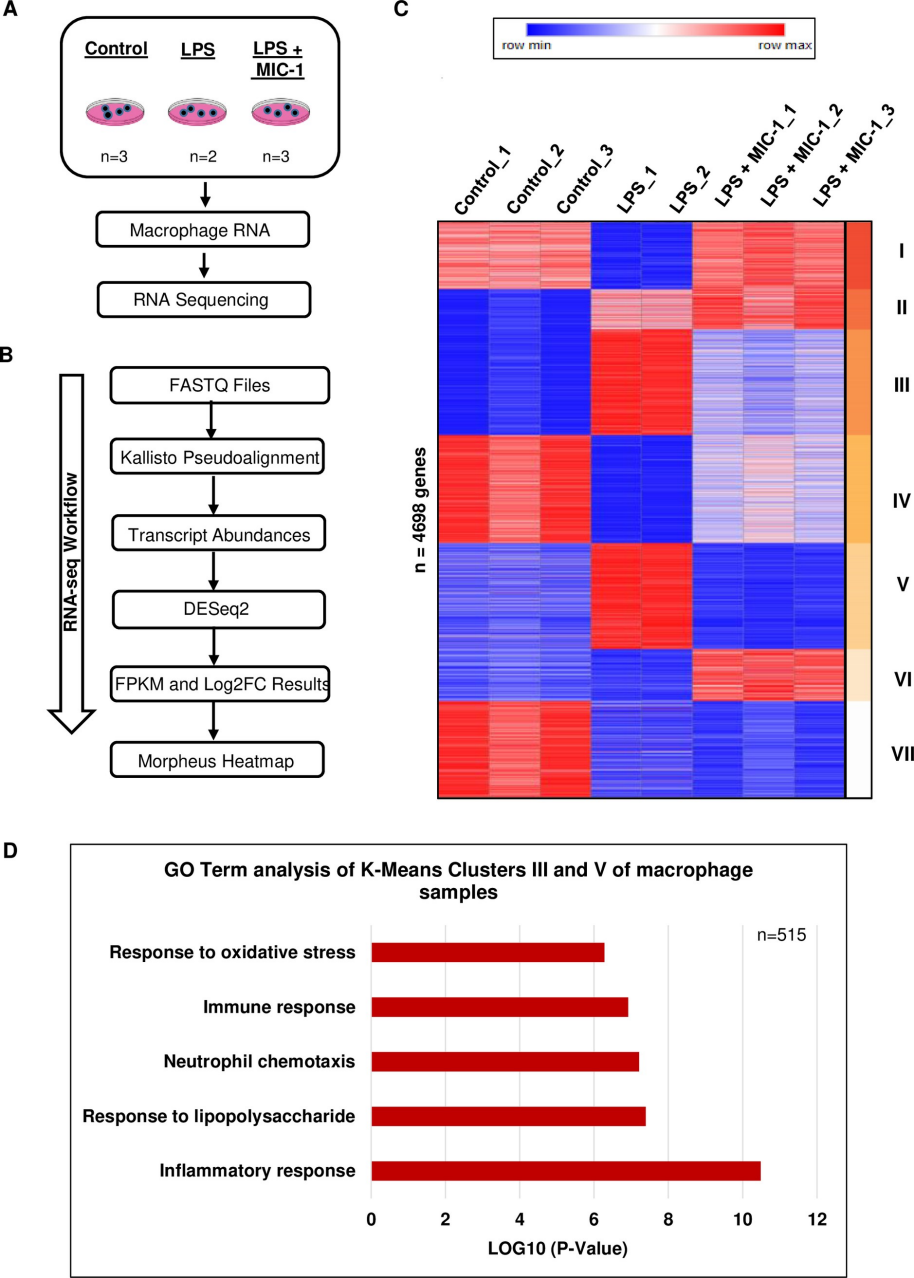
Transcriptome analysis of RAW264.7 murine macrophages. (A) Schematic workflow of RNA sequencing with control, lipopolysaccharide (LPS), LPS+10 μM MIC-1 samples. (B) RNA-seq workflow of transcriptome analysis on Raw 264.7 macrophages. (C) Transcript abundances were obtained from eight Raw 264.7 mouse macrophage samples with three control replicates, two LPS replicates, and three LPS replicates treated with 10 μM of MIC-1. DESeq2 v1.20.0 was used to generate Log2FC values of the following treatments using the transcript abundances: control vs. LPS, control vs. LPS+10 μM MIC-1, and LPS vs. LPS+10μM MIC-1. An FPKM table was outputted with the intersection, removing duplicates, of genes filtered for the absolute value of Log2FC > 1, average FPKM value per gene > 1, and adjusted P-value < 0.01 from the three aforementioned Log2FC comparisons. Using Morpheus, a heat mapping software provided by the Harvard Broad Institute, seven k-means clusters were obtained from the gene list of 4,698 genes based on the same FPKM table adjusted through z-score standardization and Euclidean distance correlation. (D) Gene ontology analysis using DAVID v6.8 functional annotations (biological process) was conducted on genes (n = 515) from k-means clusters III and V of the heatmap in Fig 1C.

To understand the molecular mechanisms of MIC-1 action, we performed a transcriptome analysis in Raw 264.7 murine macrophages treated with LPS, LPS + MIC-1, and solvent controls (Fig 1A). A differential expression analysis was conducted with DEseq2 for each pairwise sample group comparison, and the resulting genes that exhibited an absolute value of Log2FC > 1, average fragments per kilobase reads per million (FPKM) value per gene > 1, and adjusted P-value < 0.01 were further considered for k-means clustering analysis to identify clusters of genes exhibiting common responses to these treatments (Fig 1B). Seven k-means clusters were obtained from this list of 4,698 genes. Clusters III and V were particularly interesting, as these 515 genes were activated in the LPS treatment and subsequently repressed in the MIC-1 treatment (Fig 1C). Gene ontology (GO) analysis of these MIC-1 repressed genes are consistent with MIC-1 suppressing the immune response to LPS treatment, response to lipopolysaccharides, neutrophil chemotaxis, and oxidative stress (Fig 1D). These results suggest that MIC-1 decreases inflammation via inflammation and immunity and oxidative stress pathways in LPS-induced macrophages.

### MIC-1 inhibits LPS-induced acute inflammation in mice

**Fig 2:**
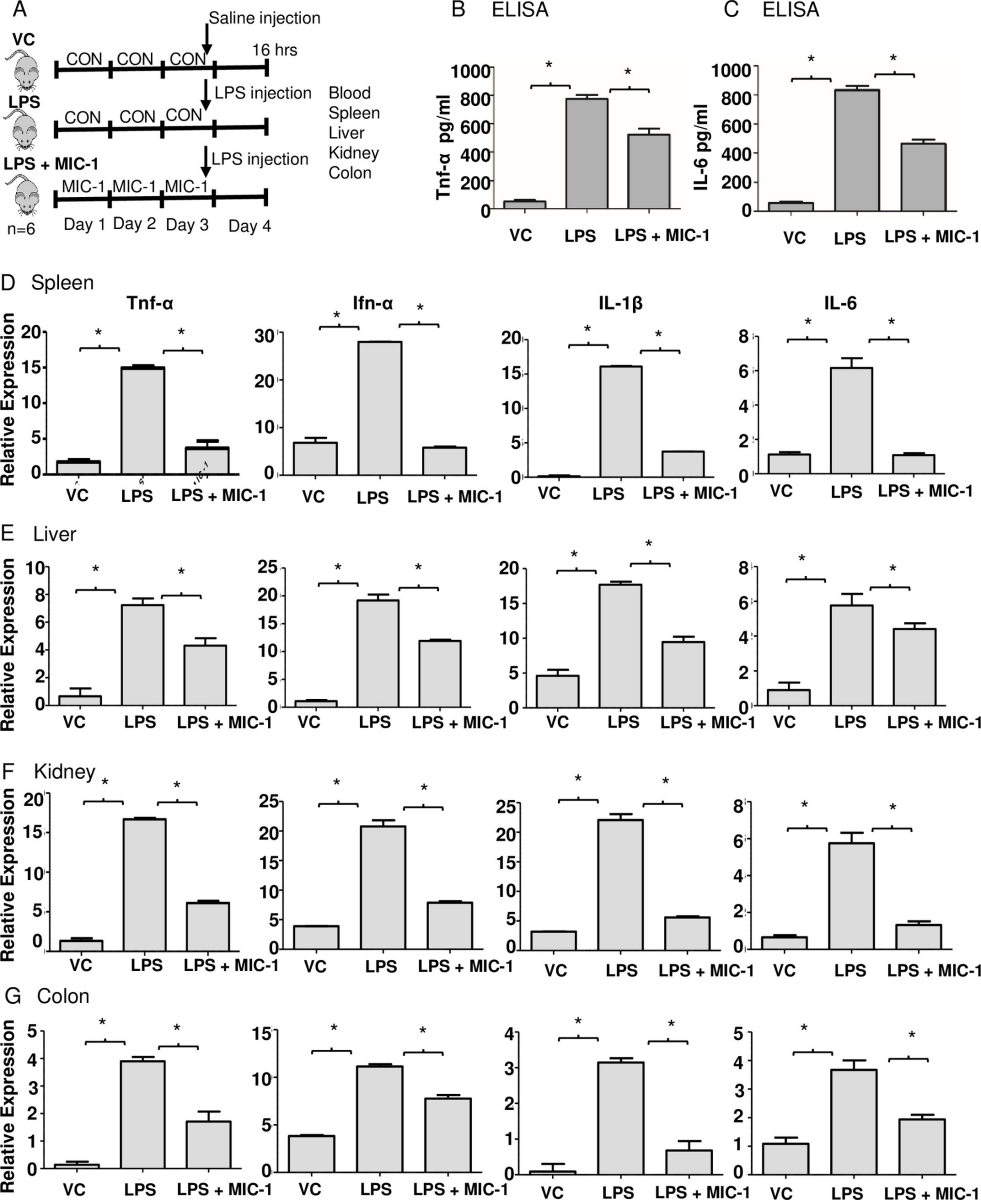
MIC-1 ameliorates LPS-induced sepsis/inflammation. (A) Schematic representation of the experimental design. Male C57BL/6 mice (n  =  18) were randomly divided into three groups: vehicle control (10% DMSO); LPS (10 mg/kg); LPS (10 mg/kg) + MIC-1 (80 mg/kg). LPS or saline control treatment was given after 3 d of gavaging MIC-1, and blood and organs collected after 16 h post LPS administration (n = 6). The levels of serum Tnf-α (B) and IL-6 (C) were determined using ELISA kits (n = 6). Tnf-α, Ifn-α, IL-1β, and IL-6 mRNA levels in the spleen (D), liver (E), kidney (F), and colon (G) measured with qPCR. Error bars indicate ± S.D. (n = 6). * indicates the data was significant at *P* ≤ 0.05.

Mice were gavaged with 80 mg/kg MIC-1 or vehicle control for three days before the intraperitoneal injection of LPS (10 mg/kg) or saline solution and sacrificed 16 h post-injection, when their blood, livers, kidneys, spleens, and colons were collected (Fig 2A). MIC-1 decreased tumor necrosis factor-alpha (Tnf-α) and interleukin 6 (IL-6) levels in LPS-induced blood serum by 1.5 and 1.8-fold, respectively (Fig 2B and 2C). Spleen enlargement caused by the activation of the immune system is a common manifestation of bacterial sepsis [35]. MIC-1 reduced the spleen weight in the LPS-treated mice by 43% compared to the LPS treatment alone (S1A and S1B Fig). MIC-1 decreased the expression of Tnf-α (75%), Ifn-α (80%), IL-1β (82%), and IL-6 (76%) in LPS-induced spleen tissue (Fig 2D). The expression of Tnf-α (40%), Ifn-α (38%), IL-1β (46%), and IL-6 (24%) was also decreased in LPS-induced liver tissue in the MIC-1 treatment (Fig 2E). MIC-1 also decreased the Tnf-α (64%), Ifn-α (63%), IL-1β (75%), and IL-6 (77%) expression in LPS-induced kidney tissue (Fig 2F) and decreased the Tnf-α (56%), Ifn-α (30%), IL-1β (78%), and IL-6 (48%) expression in LPS-induced colon tissue (Fig 2G). Overall, MIC-1 treatment was effective in suppressing the LPS-induced expression of inflammatory markers (Tnf-α, Ifn-α, IL-1β, IL-6) in the spleen, liver, kidney, and colon (Fig 2D–2G). These results indicate that MIC-1 systemically suppresses inflammation and sepsis-associated markers in the LPS-treated mice.

### Transcriptome analysis of MIC-1 effect in the LPS-induced liver

**Fig 3:**
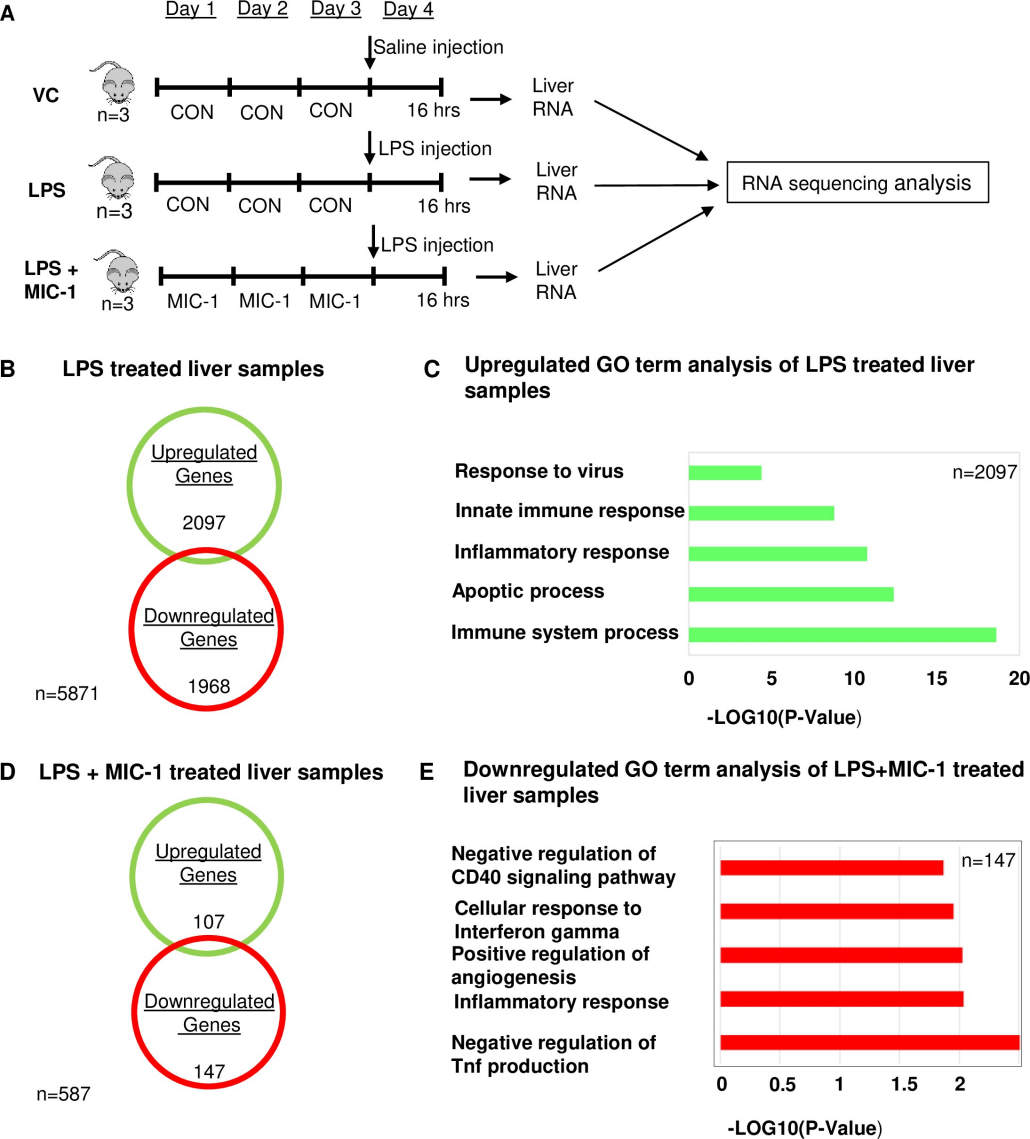
Transcriptome analysis in the liver. (A) Schematic representation of the course of the experiment. Male C57BL/6 mice (n  =  18) were divided into three groups: a. vehicle control (10% DMSO), b. LPS (10 mg/kg), c. LPS (10 mg/kg) + MIC-1 (80 mg/kg). LPS/Saline treatment was given after 3 days of gavaging MIC-1; the liver was collected after 16 hours for RNA sequencing (n = 6). (B) Venn diagram displaying the number of inducible or repressible (P-value < 0.05 and > 1.0 absolute log2-fold change) genes after LPS treatment in the liver. (C) Gene Ontology analysis using DAVID v6.8 functional annotations (biological process) up-regulated by LPS treatment only in liver samples (P-value < 0.05, log2-fold change > 1.0). (D) Venn diagram displaying the number of inducible or repressible (P-value < 0.05 and > 1.0 absolute log2-fold change) genes after LPS+MIC-1 treatment in the liver. (E) Gene Ontology analysis using DAVID (version 6.8) functional annotations (biological process) down-regulated by LPS+MIC-1 treatment in liver samples (P-value < 0.05, log2-fold change < -1.0).

The liver plays a major role in host response to sepsis, participating in the clearance of the infectious agents and breakdown products (17). Accordingly, liver dysfunction induced by sepsis contributes to disease severity (17, 18). We performed a transcriptomic analysis of liver tissue (Fig 3A) obtained from nine mice: three controls, three LPS-treated, and three LPS + MIC-1-treated. We focused on two comparisons to understand the response to LPS and the potential activities of MIC-1 in this tissue: 1) control samples vs LPS-treated samples and 2) LPS-treated samples versus LPS + MIC-1-treated samples. We found 2,097 upregulated genes (P-value < 0.05 and > 1.0 absolute log2-fold change) after LPS treatment (Fig 3B). GO analysis of these upregulated genes revealed that the most enriched and meaningful biological process terms were, as expected, involved in functions such as immune-inflammation response (Fig 3C). A total of 107 upregulated and 147 downregulated genes were identified in the MIC-1 + LPS-treated compared to LPS-treated samples, respectively (P-value < 0.05 and > 1.0 absolute log2-fold change) (Fig 3D). GO analysis showed that annotations down-regulated in the MIC-1 + LPS treatment were associated with inflammatory responses and negative regulation of the production of tumor necrosis factor in the liver (P-value < 0.05, log2-fold change < -1.0) (Fig 3E). These results support the hypothesis that MIC-1 protects LPS-exposed mouse liver by reducing the acute inflammatory response.

### MIC-1 may reduce oxidative stress and inflammation by promoting Nrf2 nuclear transport

**Fig 4:**
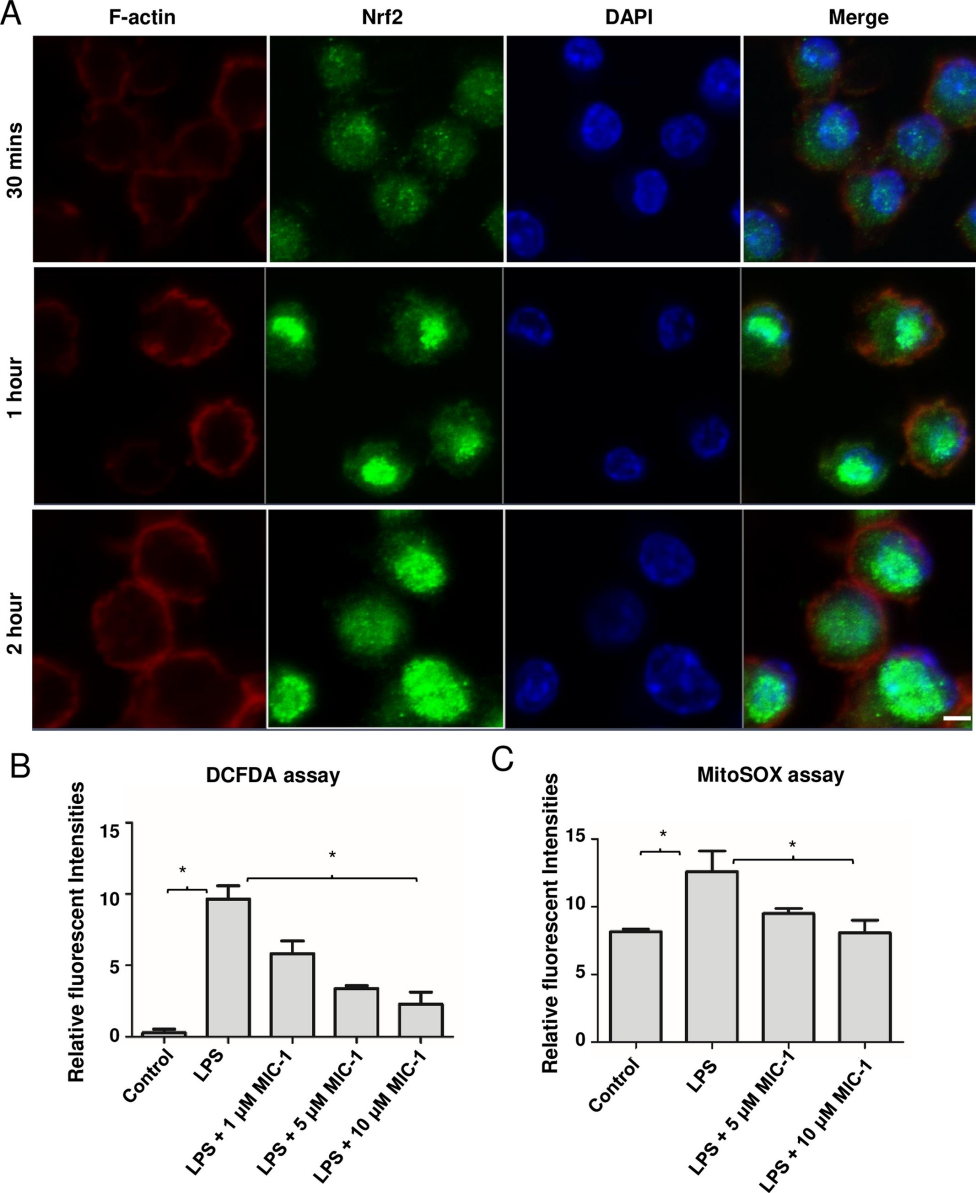
MIC-1 increases Nrf2 nuclear accumulation. (A) Raw 264.7 macrophages were treated with 1 μM MIC for 30 min, 1 h, and 2 h. Immunocytochemistry was performed with fixed cells stained with Nrf2 antibodies (green fluorescence), DAPI (blue fluorescence), and F-actin antibodies (red fluorescence). Scale bars- 5μm. (B) Effect of MIC-1 on intercellular ROS in macrophage cells induced by LPS. After the cells were treated with control, LPS, and LPS + MIC-1 for 24 h, intracellular ROS were quantified with DCFHDA dye. Error bars indicate ± S.D. (n = 3). * indicates the data was significant with P-value ≤ 0.05. (C) Effect of MIC-1 on mitochondrial superoxide in cells induced by LPS. Cells were treated with LPS, and LPS + MIC-1 and vehicle control for 24 h and mitochondrial damage was accessed with MitoSOX red dye. Error bars indicate ± S.D. (n = 3). * indicates the data was significant with P ≤ 0.05.

Previously, we demonstrated that MIC-1 is a potent activator of Nrf2-regulated genes, believed to be the target of structurally-related Brassicaceae ITCs [7]. For most of the studied genes, MIC-1 was effective at low μM and high nM levels. To provide a mechanistic explanation for this activation, and to demonstrate that MIC-1 enhances nuclear translocation of Nrf2, murine macrophages were treated with 1 μM MIC-1 for 30 min, 1 h, and 2 h. Immunocytochemistry was performed in fixed cells reacted with Nrf2 antibodies and stained with green fluorescing secondary antibodies and F-actin antibodies (red fluorescence). Nuclei were visualized with DAPI staining (blue fluorescence). MIC-1 enhanced nuclear accumulation of Nrf2 (Fig 4A), and was visible 1 h after treatment, becoming stronger after 2 h. Time-dependent increase in the Nrf2-associated fluorescence in the nuclei of the MIC-1-treated cells may also indicate that MIC-1 increases the total cellular content of Nrf2.

### Anti-oxidative stress activity of MIC-1 in mitochondria and cytoplasm

Nrf2 is a well-known master regulator of anti-oxidative responses and suppresser of cellular and mitochondrial damage caused by the overproduction of superoxide and other ROS [36, 37]. Therefore, we examined the effect of MIC-1 on the intracellular content of ROS and mitochondrial superoxide production in murine macrophages undergoing oxidative stress induced by LPS [38]. Using 2’,7’, dichlorofluorescein diacetate (DCDFA), which reacts with ROS in the cytoplasm, we observed that 10 μM MIC-1 decreased ROS levels in the LPS-treated cells by 76% (Fig 4B). We also evaluated the effect of MIC-1 on mitochondrial superoxide content (MitoSOX assay) and mitochondrion membrane potential (Rodamine123 assay). Mitochondrial superoxide-associated fluorescence was reduced by 46% in the LPS-induced cells treated with 10 μM MIC-1 compared to LPS-treated cells alone (Fig 4C). MIC-1 also restored LPS-reduced mitochondrial membrane potential to the levels observed in non-treated controls (S2 Fig), documenting its strong protective effect on the oxidative stress in mitochondria.

### MIC-1 inhibits the NF-κB nuclear transport and proinflammatory gene transcription

**Fig 5:**
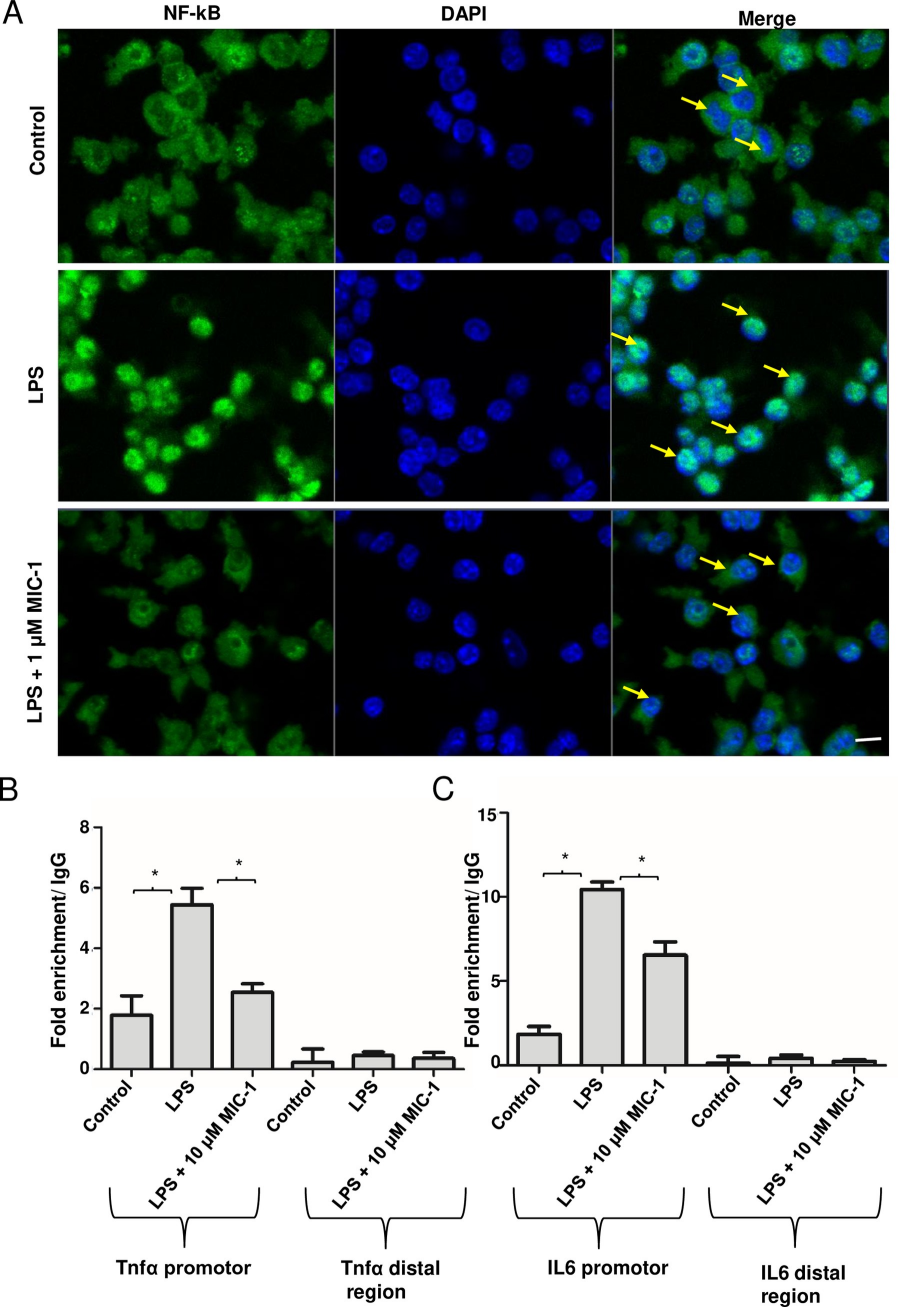
MIC-1 inhibits the NF-κB pathway. (A) The effects of MIC-1 on LPS-induced nuclear translocation of NF-κB in RAW 264.7 macrophage cells. The translocation of NF-κB (p65) to the nucleus was analyzed by confocal microscopy. Macrophages were immunostained using FITC for NF-κB and DAPI to label nuclei. Treatments: Control, LPS; LPS + 1 μM MIC-1 for 3 h. MIC-1 decreased the translocation of NFkB after LPS treatment. Scale bars- 20 μm. (B) Binding of NF-κB/p65 to the promoters of Tnf-α and IL-6 genes. ChIP assays were performed to measure the binding of NF-κB to Tnf-α and IL-6 promoters after LPS and treatment with MIC-1 using specific antibodies. *P < 0.05, two-tailed test; (n = 3).

NF-κB, a heterodimer composed of p65 and p50, is a crucial transcriptional regulator of inflammation [39]. Our previous studies showed that MIC-1 can downregulate NF-κB responsive genes, such as IL-6 and Tnf-α, as well as intestinal and subcutaneous inflammation [7, 10]. To test whether MIC-1 downregulates proinflammatory genes by directly affecting nuclear translocation of NF-κB, we performed immunocytochemical staining with the NF-κB antibody on untreated and LPS-induced macrophages with and without MIC-1 (Fig 5A). Immunofluorescence confocal microscopy revealed that an increase in nuclear translocation of NF-κB in LPS-treated cells was inhibited by MIC-1 within 3 h. While LPS treatment increased total NF-κB levels in the cells, MIC-1 mitigated this increase to the levels of the untreated cells. We also investigated whether MIC-1 decreases specific binding of NF-κB to the promoter regions of the NF-κB-regulated genes, such as Tnf-α and IL-6, in LPS-induced macrophages using a chromatin immunoprecipitation (ChIP) assay. ChIP has been developed for studying protein–DNA interactions that can be subsequently quantified using qPCR. As expected, the NF-κB gene displayed significantly stronger binding to a Tnf-α promoter (3-fold) and IL-6 promoter (5-fold) in LPS-treated cells (Fig 5B and 5C). This binding was reduced by 2-fold for Tnf-α and 1.5-fold for IL-6 promoter in MIC-1-treated cells.

## Discussion

This manuscript reports on two mutually complementary, novel, and clinically important experimental observations. Firstly, it documents powerful anti-oxidative stress and anti-inflammatory effects of MIC-1 in a clinically relevant mouse model with LPS-induced sepsis and acute inflammation, and *in vitro*. Secondly, it elucidates molecular and genetic mechanisms behind this effect through transcriptomics analysis, immunocytochemical confirmation of the MIC-1-stimulated nuclear accumulation of Nrf2, and inhibition of nuclear accumulation of NF-κB in the LPS-induced murine macrophages. At 1 μM, MIC-1 enhanced Nrf2 and inhibited NF-κB accumulation in the nucleus of macrophages treated with LPS; this effect was observed within 3 h of treatment (Figs 4 and 5).

Our understanding of the molecular mechanisms behind anti-oxidative stress and anti-inflammatory action of ITCs is still incomplete. This is particularly true for newly isolated, ring glycosylated moringa ITCs, such as MIC-1, that are more stable than Brassicaceae ITCs and often more efficacious. All ITCs are powerful electrophiles that are effective in preventing or reducing inflammation [40, 41]. They are thought to facilitate oxidation or conjugation of cysteine residues (e.g., Cys^151, 273, 288^) on the protein Kelch-like ECH-associated protein 1 (Keap1) [42]. Keap1 is one of the principal regulators of Nrf2 activity in cells; it facilitates the polyubiquitination of Nrf2, thereby enabling its proteasomal degradation [43]. C151 is one of four cysteine residues preferentially modified by broccoli sulforaphane resulting in Nrf2 accumulation in the cytoplasm and its enhanced translocation into the nucleus, leading to the upregulation of ARE genes that protect against ROS and xenobiotic electrophiles [44]. NF-κB plays a crucial role in inflammation due to its involvement in cell growth, proliferation, angiogenesis, invasion, apoptosis, and survival [45]. Some Brassicaceae ITCs were shown to prevent the degradation of the NF-κB inhibitor, IκB [46], thus reducing nuclear translocation of NF-κB and its transcriptional activation of the pro-inflammatory cascade [19, 47, 48].

The transcriptomic analysis of the effect of MIC-1 on the LPS-induced macrophages confirm its ability to activate the anti-inflammatory, anti-oxidative stress, and immunosuppressive pathways (Fig 1). These results translated into systemic improvements in the *in vivo* oxidative stress and inflammatory markers and pathways in the mouse LPS-induced sepsis / acute inflammation model (Figs 2 and 3). LPS-induced sepsis simulates the pathological process of systemic inflammation and acute hepatitis resulting from sepsis-associated endotoxemia [49]. Activated by LPS, macrophages produce and release chemokines and proinflammatory cytokines that mediate the migration of macrophages and cause a cascade of events leading to inflammatory injury and organ failure [1]. This explains a significant increase in Tnf-α, Ifn-α, IL-1β, and IL-6 mRNAs in the LPS stimulated spleen, liver, and kidney and demonstrates the effective suppression of these key sepsis markers by MIC-1 (Fig 2). We focused our comprehensive transcriptomic analysis on the liver, since liver dysfunction may severely disrupt immunological homeostasis in critically ill patients and frequently promotes progression to multi-organ failure [50, 51]. We further checked whether MIC-1 decreases LPS induced inflammation in human monocytes. The expression of IL-6 (70.5%), INOS (95%) and INOS (68.75%) was decreased in LPS-induced human monocytes in the 10 uM MIC-1 treatment (S3 Fig). Our analysis confirmed the ability of MIC-1 to mitigate gene expression pathways associated with the pathological effects of sepsis and acute inflammation (Fig 3). Moreover, spleen enlargement (splenic hypoplasia) is a common and rapidly manifested clinical side effect of sepsis [35]. The reversal of LPS-induced splenic hypoplasia by MIC-1 to the levels observed in healthy animals (S1A and S1B Fig) compliments the gene expression data and supports development of MIC-1 as anti-inflammatory and anti-sepsis medication. We have not used MIC-1 as a control in these experiments, since it was already reported to show similar effects as MIC-1 treated LPS samples [6]. We performed transcriptomic analysis on the liver for this study, but for our future studies peritoneal macrophages will be used along with other organs which are collected in this study.

In addition to documenting the pharmacological effectiveness of MIC-1 in mice, this manuscript is the first to directly demonstrate that MIC-1 increases nuclear accumulation of Nrf2 and decreases nuclear accumulation of NF-κB, which are major transcription factors involved in oxidative stress and inflammation responses. It is tempting to speculate that this observation also extends to structurally related Brassicaceae ITCs. These data provide a direct mechanistic link to the observed *in vitro* and *in vivo* activities of MIC-1. In addition, we were able to demonstrate the downstream effects of Nrf2 activation, such as reduction of ROS in mitochondria and cytoplasm of the LPS-induced murine macrophages (Fig 4B and 4C). Furthermore, using a ChIP assay, we showed that MIC-1 decreases NF-κB binding to the promoter regions of two key proinflammatory cytokines: Tnf-α and IL-6 (Fig 5B and 5C).

**Fig 6:**
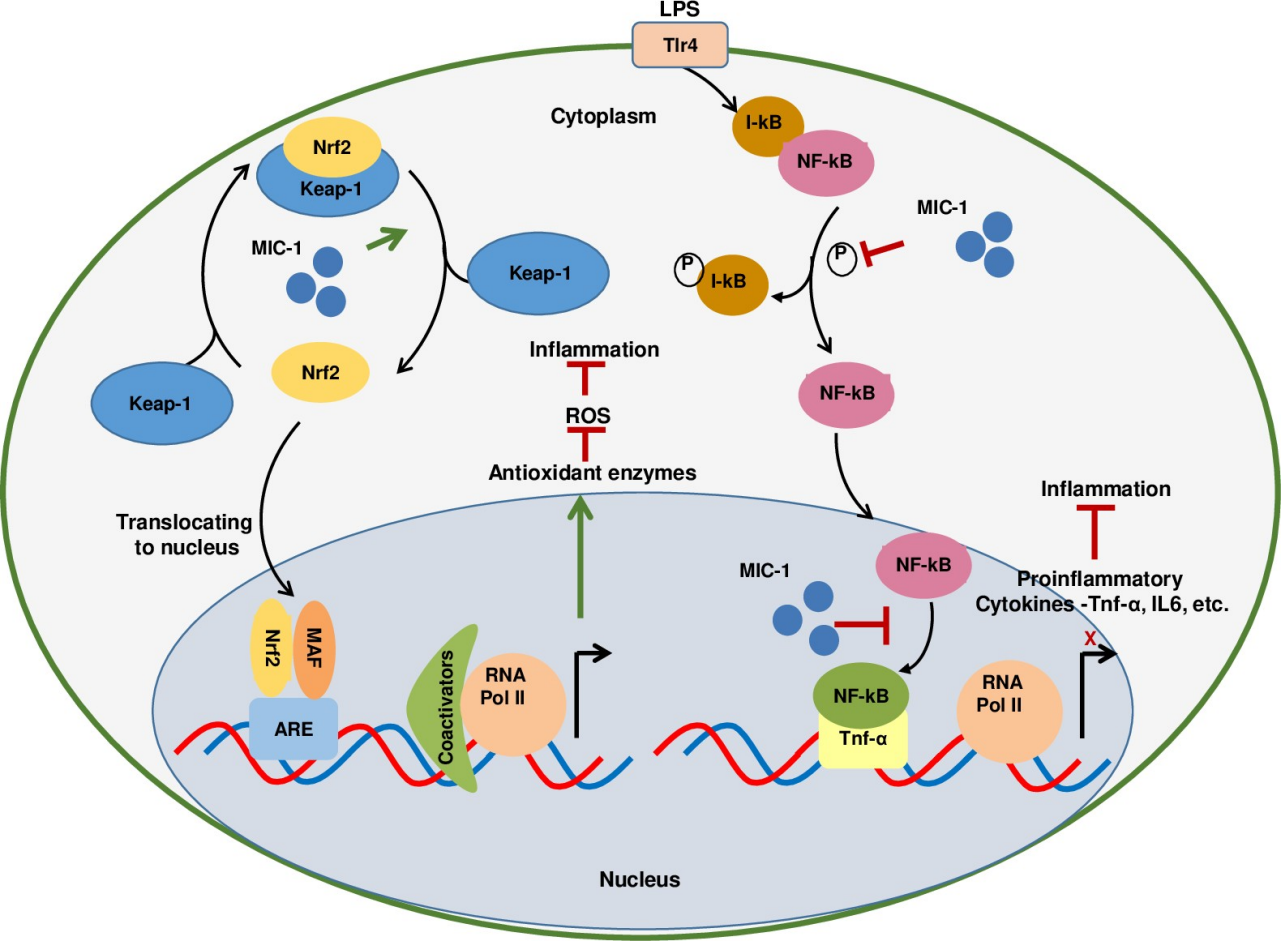
A schematic model of the hypothesized mechanisms of MIC-1 molecular action affecting Nrf2 and NF-kB pathways. MIC-1 increases the cellular content and nuclear translocation of Nrf2 by releasing it from the Keap-1 complex resulting in the heightened expression of Nrf2 target genes coding for antioxidant enzymes. MIC-1 also prevents the phosphorylation of the NF-κB inhibitor, Iκb, and nuclear translocation of NF-κB, thus suppressing the transcription of pro-inflammatory genes. ARE, antioxidant response element; Iκb, an inhibitor of kappa B; Keap1, Kelch-like ECH-associated protein1; MAF, Nrf2 transcriptional factor; Nrf2, nuclear factorE2-related factor 2; NF-κB, nuclear factor kappa B, RNA Pol II, RNA polymerase II; ROS, reactive oxygen species.

Our data suggests that MIC-1 affects early transduction stages of the Nrf2 anti-oxidant and NF-κB anti-inflammatory signaling, which are the key processes contributing to the etiology of many diseases. Simultaneous transcriptional activation of the Nrf2 pathway and downregulation of the NF-κB pathway by one compound has not been reported previously. The proposed mechanism of action of MIC-1, whereby it simultaneously activates Nrf2 and inhibits NF-κB signaling is summarized in Fig 6, although some elements of the model require further conformation through future research. Our data do not allow us to exclude the possibility that MIC-1 affects Nrf2 and NF-κB signaling indirectly, via some yet uncharacterized modulator, rather than through direct interaction with Nrf2 and NF-κB complexes. In addition, we cannot exclude the crosstalk between Nrf2 and NF-κB pathways. Nevertheless, our data provides the foundation for further clinical development of MIC-1 as a novel pharmaceutical agent for inflammation-related diseases.

## Supporting information

S1 Fig MIC-1 reduces inflammation in the spleen in vivo. Reduced spleen size after treating with MIC-1 after LPS induced inflammation or sepsis. Images of the spleen (A) and weight of the spleen (n = 6) (B).
(TIF)

S2 Fig Effects of MIC-1 on mitochondrial membrane potential. Effect of MIC-1 on mitochondrial membrane potential in macrophage cells induced by LPS. After the cells were treated with the control, LPS, and LPS + MIC-1 for 24 hours, the mitochondrial membrane potential was assessed with the fluorescent dye Rh123. Relative fluorescence intensity was measured and expressed in arbitrary units (a.u.). Error bars indicate ± S.D. (n = 3). * indicates the data was significant at P ≤ 0.05.
(TIF)

S3 Fig MIC-1 inhibits LPS-induced inflammation in human monocytes. MIC-1 decreased the expression of IL-6, INOS and INOS and in LPS-induced human monocytes. Error bars indicate ± S.D. (n = 6). * indicates the data was significant at P ≤ 0.05.
(TIF)

S1 Table Primer sequences used in the RT PCR and ChIP analysis. (TIF)
