# Supplementary figures and images for: Moringa isothiocyanate-1 regulates Nrf2 and NF-κB pathway in response to LPS-driven sepsis and inflammation
Source: PLoS One. 2021 Apr 1;16(4):e0248691. doi: 10.1371/journal.pone.0248691 (PMC8016325; doi:10.1371/journal.pone.0248691)

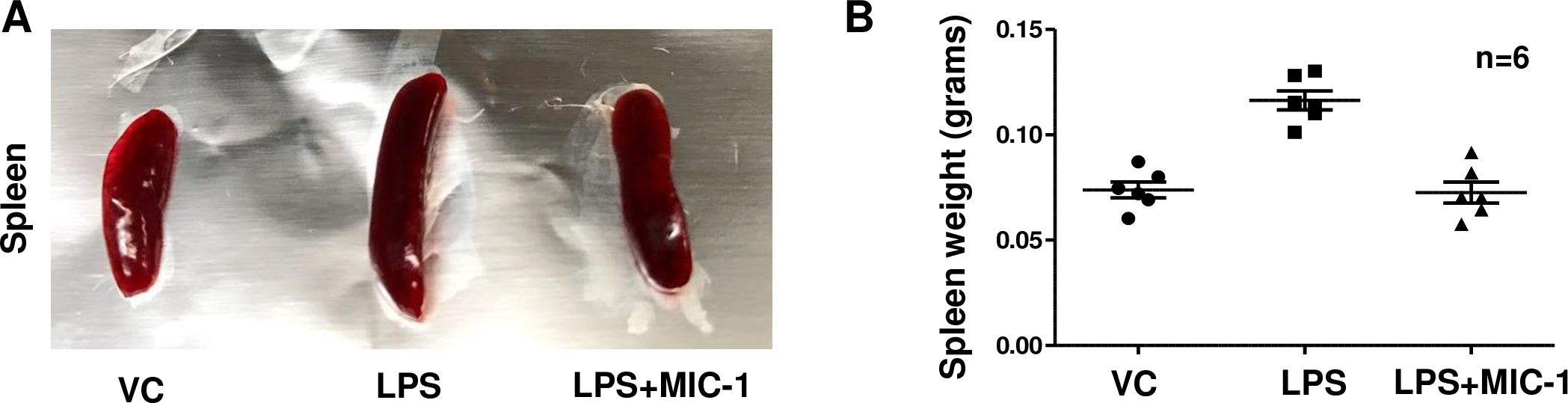

Supplement: S1 Fig — Reduced spleen size after treating with MIC-1 after LPS induced inflammation or sepsis. Images of the spleen (A) and weight of the spleen (n = 6) (B). (TIF) [file pone.0248691.s001.tif]

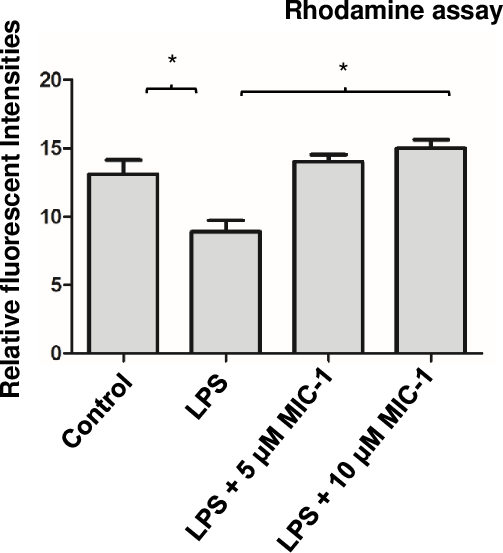

Supplement: S2 Fig — Effect of MIC-1 on mitochondrial membrane potential in macrophage cells induced by LPS. After the cells were treated with the control, LPS, and LPS + MIC-1 for 24 hours, the mitochondrial membrane potential was assessed with the fluorescent dye Rh123. Relative fluorescence intensity was measured and expressed in arbitrary units (a.u.). Error bars indicate ± S.D. (n = 3). * indicates the data was significant at P ≤ 0.05. (TIF) [file pone.0248691.s002.tif]

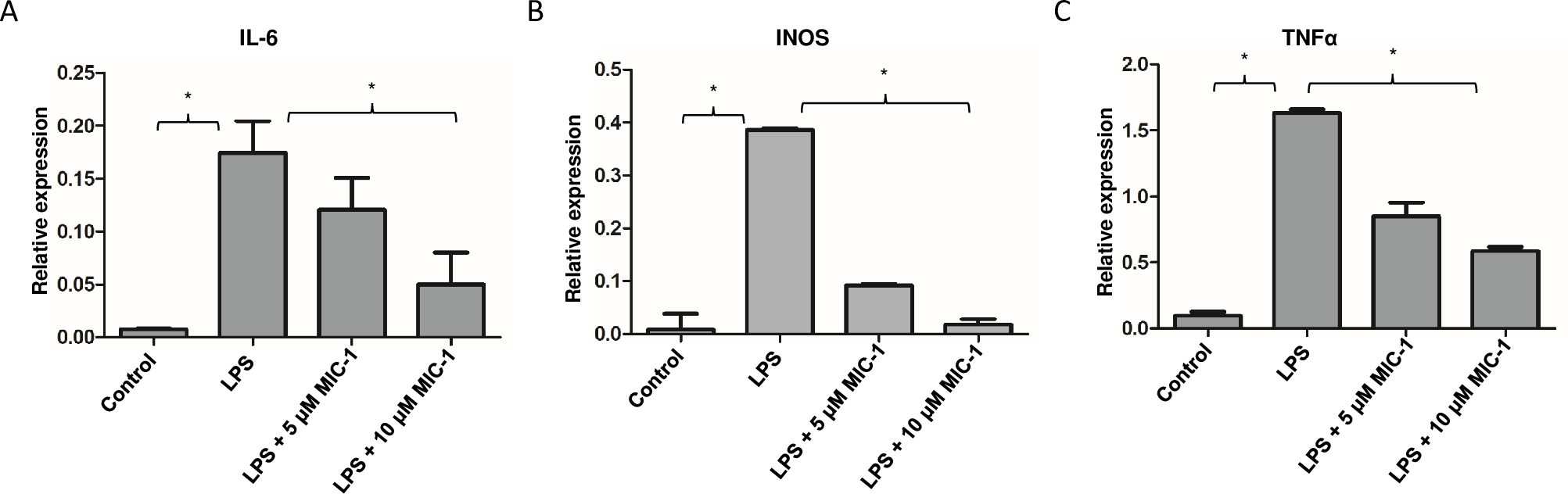

Supplement: S3 Fig — MIC-1 decreased the expression of IL-6, INOS and INOS and in LPS-induced human monocytes. Error bars indicate ± S.D. (n = 6). * indicates the data was significant at P ≤ 0.05. (TIF) [file pone.0248691.s003.tif]

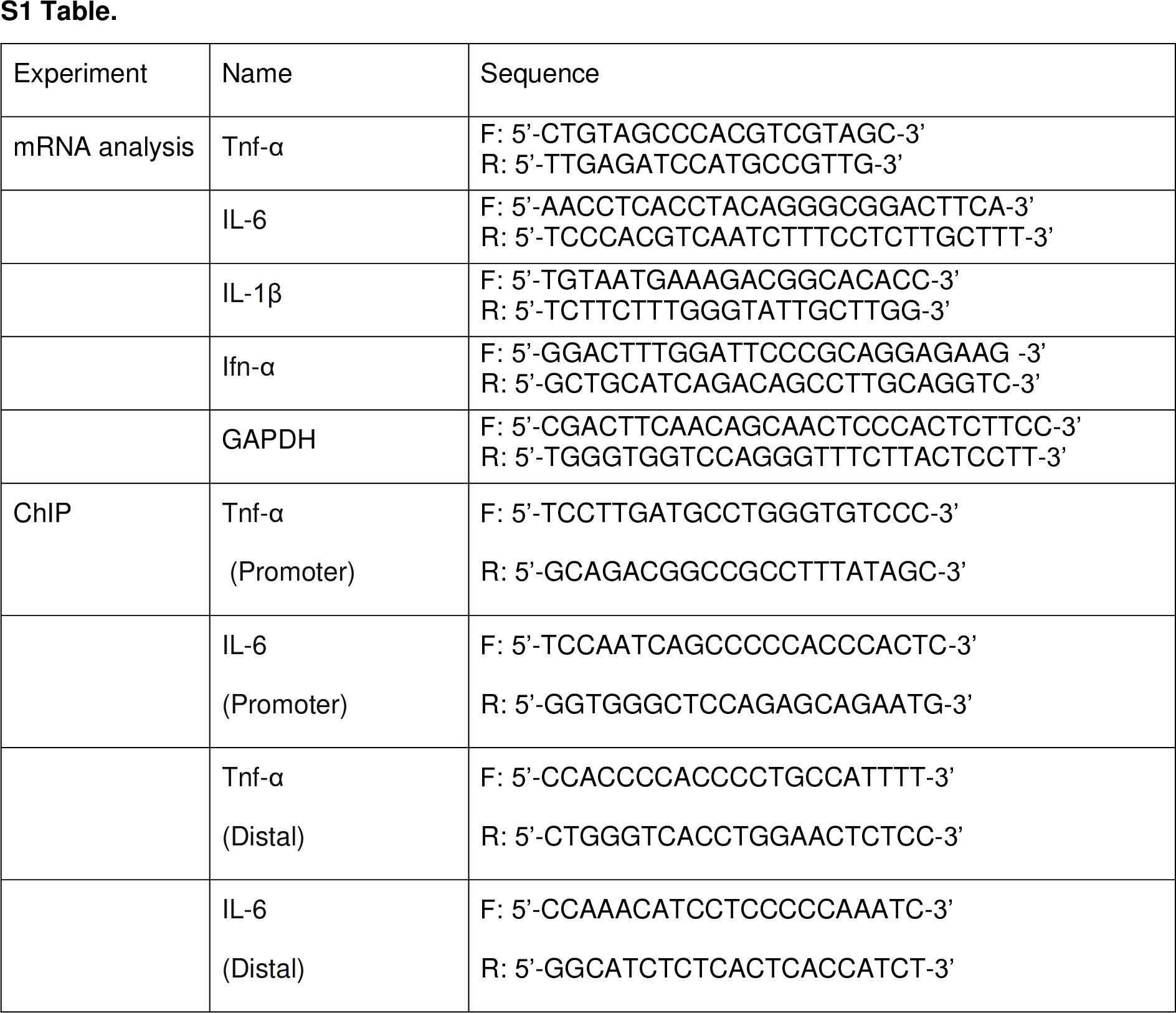

Supplement: S1 Table — (TIF) [file pone.0248691.s004.tif]
